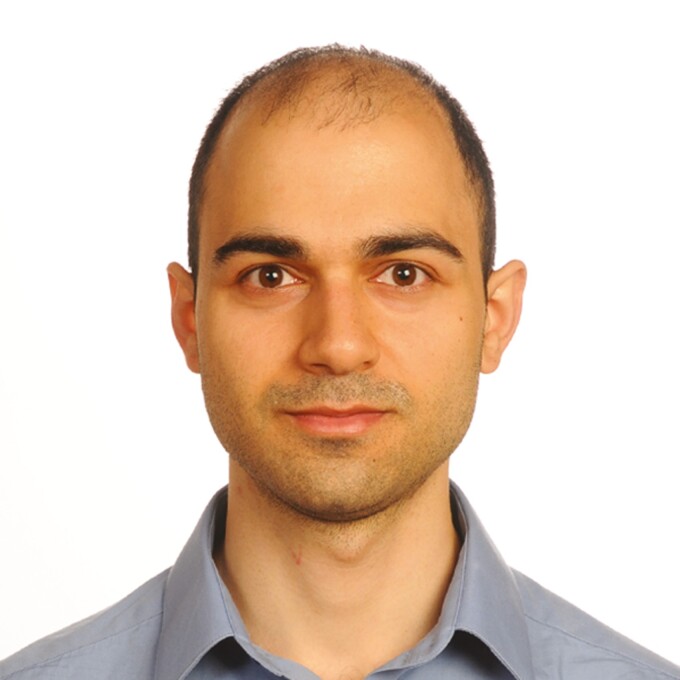# Crohn’s & Colitis 360 Editorial Fellowship: The Inaugural Fellow’s Insights

**DOI:** 10.1093/crocol/otae002

**Published:** 2024-01-31

**Authors:** Gassan Kassim

**Affiliations:** Division of Gastroenterology and Hepatology, Department of Internal Medicine, Mayo Clinic Arizona, Scottsdale, AZ, USA

The fields of medicine, gastroenterology (GI), and even more specifically, inflammatory bowel disease (IBD) are evolving at an unprecedented rate. As fellows in training and as physicians, it is our responsibility not only to be well versed in the fundamentals of our field but also to be aware of the most up-to-date scientific discussions and discoveries. Fulfilling both responsibilities is critical to allow for effective evidence-based patient care.

Over the past 5–10 years, numerous editorial fellowships have been created within the field of GI to help facilitate fellow engagement in research and to educate fellows about the intricacies of the editorial process.^1–3^ The Crohn’s and Colitis Foundation currently has 2 peer-reviewed, IBD-focused journals, *Inflammatory Bowel Diseases* and *Crohn’s & Colitis 360*, both of which have their own editorial fellowships. Participants in the Inflammatory Bowel Diseases Journal Editorial Fellowship recently published their experiences.^1^ In this training pearl, we aim to highlight the Crohn’s & Colitis 360 Editorial Fellowship from the perspective of the inaugural editorial fellow and demonstrate how it equips fellows with the tools needed to create awareness of and contributions to cutting-edge scholarship in the field of IBD.

The Crohn’s & Colitis 360 Editorial Fellowship is a year-long fellowship during which a GI or advanced IBD fellow gets a front-row seat to the inner workings of a peer-reviewed journal and actively participates in the editorial process. The fellow is paired with 2 assigned associate editors (AEs) with whom they meet monthly.

At the start of the program, the editorial fellow is provided an evidence-based Reviewer Guide containing a comprehensive list of resources explaining best practices in the peer-review process. The fellow reviews these materials and then discusses with the AEs. To apply this knowledge, the fellow is then assigned manuscripts for review and subsequently drafts their feedback prior to each meeting with the AEs. During the meetings, a critical review of the manuscript is conducted with a focus on the background of the paper, its relevance in the current literature landscape, the methodology, results, discussion, and conclusion. The fellow revises their review based on this discussion and submits their review through the journal’s online review portal. The review is evaluated by the journal’s Deputy Editor or Editor in Chief, a final decision is made, and the peer reviews are sent to the corresponding author.

As the inaugural editorial fellow of the *Crohn’s & Colitis 360* journal, I would like to share 5 personal highlights of my experience to encourage future fellows to strongly consider this unique opportunity.

Being immersed in IBD literature. Every month I was assigned a manuscript to read and review. Papers reviewed were diverse and varied broadly in topics from IBD medical education to real-world experience with newer therapeutic agents to the management of extraintestinal manifestations. A deeper dive into each question through a literature review was typically part of the process. Directly and indirectly, this allows the fellow to keep their finger on the pulse of the field and identify gaps that might spark further academic endeavors.
Learning how to create a constructive peer review for the authors. Going through the Reviewer Guide builds a solid foundation, and regular application of this knowledge throughout the fellowship reinforces this knowledge. Not only Do the fellows learn the technical aspects of how to structure and communicate an effective, concise, and constructive peer review, they also explore the broader considerations that play into editorial decisions such as clinical relevance, context, relationships to the currently available literature, and impacts on patient care.
Unparalleled mentorship. Throughout the year, the fellow works closely with 2 exceptionally inspiring mentors who not only guide you and educate you on the editorial process, but also help grow your clinical skill set and scientific reasoning. They demonstrate an infectious passion for the field and inspire you to become the best version of yourself on countless levels.
Receiving coaching on time management. Going through this fellowship, you get to witness firsthand what proper time management means as your mentors repeatedly and subconsciously demonstrate excellent time management skills. You will work to navigate time zones, coordinate online meetings, establish deadlines, and become far more effective and productive as you simultaneously balance your clinical duties. Cultivating this skillset will serve every clinician and researcher.
Fostering love of IBD. While most fellows who apply to this program will demonstrate a baseline interest in the field, the appreciation will certainly deepen during the fellowship. Exposure to the IBD research landscape, exposure to the dedication and commitment of AEs to the field, and cultivation of greater awareness of the needs of patients with IBD undoubtedly inspire a deep and profound appreciation for the field collectively.

Overall, this has been a tremendous opportunity that elevated my general GI training to the next level. It deepened my commitment to the field, refined my skills, allowed me to create lasting relationships with my mentors, provided me with visibility within the IBD space, and boosted my career development. Ultimately, I am a better clinician and better advocate for the care of my patients. I am grateful for the Foundation for all it does, not just for advancing the field and patient care but also for the profound commitment to the education of future IBD physicians.